# A decade-long shift in use of energy devices for BABA robotic thyroidectomy: automated video analysis by deep learning

**DOI:** 10.1007/s11701-025-03113-7

**Published:** 2026-03-17

**Authors:** Hyeong Won Yu, Seoung-Ho Choi, Yoon Kong, Ja Kyung Lee, Woochul Kim, Dongheon Lee, Su-jin Kim, Young Jun Chai, June Young Choi, Kyu Eun Lee

**Affiliations:** 1 Department of Surgery, Seoul National University Bundang Hospital, 82, Gumi-ro 173 Beon-gil, Bundang-gu, Seongnam-si, Gyeonggi-do 13620 Republic of Korea; 2 Department of Surgery, Seoul National University College of Medicine, 101 Daehak-ro, Jongno-gu, Seoul, Republic of Korea; 3 Basic Liberal Arts, Hansung University College of Liberal Arts, 116 Samseongyoro-16gil, Seongbuk-gu, Seoul, Republic of Korea; 4 Department of Surgery, Seoul National University Hospital, 101 Daehak-ro, Jongno-gu, Seoul, Republic of Korea; 5 Institute of Medical and Biological Engineering, Seoul National University Medical Research Center, 101 Daehak-ro Jongno-gu, Seoul, Republic of Korea; 6 Department of Radiology, Seoul National University College of Medicine, Seoul National University Hospital, 101 Daehak-ro Jongno-gu, Seoul, Republic of Korea; 7 Department of Surgery, Seoul National University Boramae Medical Center, 20 Boramae-ro 5-gil, Dongjak-gu, Seoul, Republic of Korea

**Keywords:** BABA RT, Robotic thyroidectomy, Energy device, Localization, Deep learning

## Abstract

Bilateral axillo-breast approach robotic thyroidectomy (BABA RT) is a well-established minimally invasive surgical option; however, shifts in preferences regarding the energy devices used for the procedure have not been analyzed quantitatively. This study retrospectively reviewed 81 BABA-RT videos from 2013 to 2021, and a YOLOv5 deep learning model was used to analyze the use of four energy devices: Harmonic ACE curved shears, Permanent cautery hook, ProGrasp forceps, and Maryland bipolar forceps. The data revealed a significant temporal shift in the type of device used. Use of Harmonic ACE curved shears fell from 72.3% in 2013 to 20.7% in 2021. By contrast, use of the Permanent cautery hook increased from 27.7% to 79.3% during the same period. There was no change in use of the other two devices. This study shows that deep learning is a tool that can be used to track surgical device usage based on video evidence. The findings indicate a gradual shift in the device chosen for BABA RT, from Harmonic ACE curved shears to a Permanent cautery hook, over the past decade, and reflect evolving surgical preferences and techniques.

**Supplementary Information:**

The online version contains supplementary material available at 10.1007/s11701-025-03113-7.

## Introduction

Development of robotic platforms has expanded the scope of minimally invasive thyroid surgery [1]. Since its first clinical use in 2009, bilateral axillo-breast approach robotic thyroidectomy (BABA RT) has been adopted widely, with more than 5,000 cases reported globally [2, 3]. The BABA RT technique uses four small incisions, two in the axillae and two at the areolar margins, to create subcutaneous tunnels for the robotic arms. Usually, grasping forceps, e.g., ProGrasp or Maryland bipolar forceps, are inserted through the two axillary ports, while the right areolar port holds the camera, and the left is used for the energy device [4].

Although various energy devices are available, the BABA RT approach primarily uses Harmonic ACE curved shears or a Permanent cautery hook. These energy devices are crucial for achieving hemostasis when dissecting thyroid tissue from the surrounding structures; thus, the choice of device has a direct influence the surgical technique [5]. During the initial period in which the technique was developed (around 2009), the Harmonic ACE curved shears was the principal energy device; however, a longitudinal survey of devices used at later time points would provide an objective analysis of changes in surgical methods and trends.

Several deep learning-based localization methods have been developed to analyze videos of robot-assisted thyroidectomy and detect surgical instruments (including energy devices) automatically [6–9]; however, these models have been used primarily for intraoperative purposes (such as providing alerts that prevent instruments from entering critical anatomical areas), or for postoperative analysis (such as evaluation of a surgeon’s skill) [7, 10–12].

In this study, we investigated how the use of energy devices evolved over a 9-year period (2013–2021). To do this, we used an algorithm that automatically detects energy devices used in videos of robot-assisted thyroidectomy. Specifically, we used YOLOv5 [13], a deep learning-based object detection algorithm, to analyze BABA RT surgical videos retrospectively, identify each energy device, and provide a quantitative measure of usage frequency.

## Methods

### Study design and patient selection

**Fig. 1:**
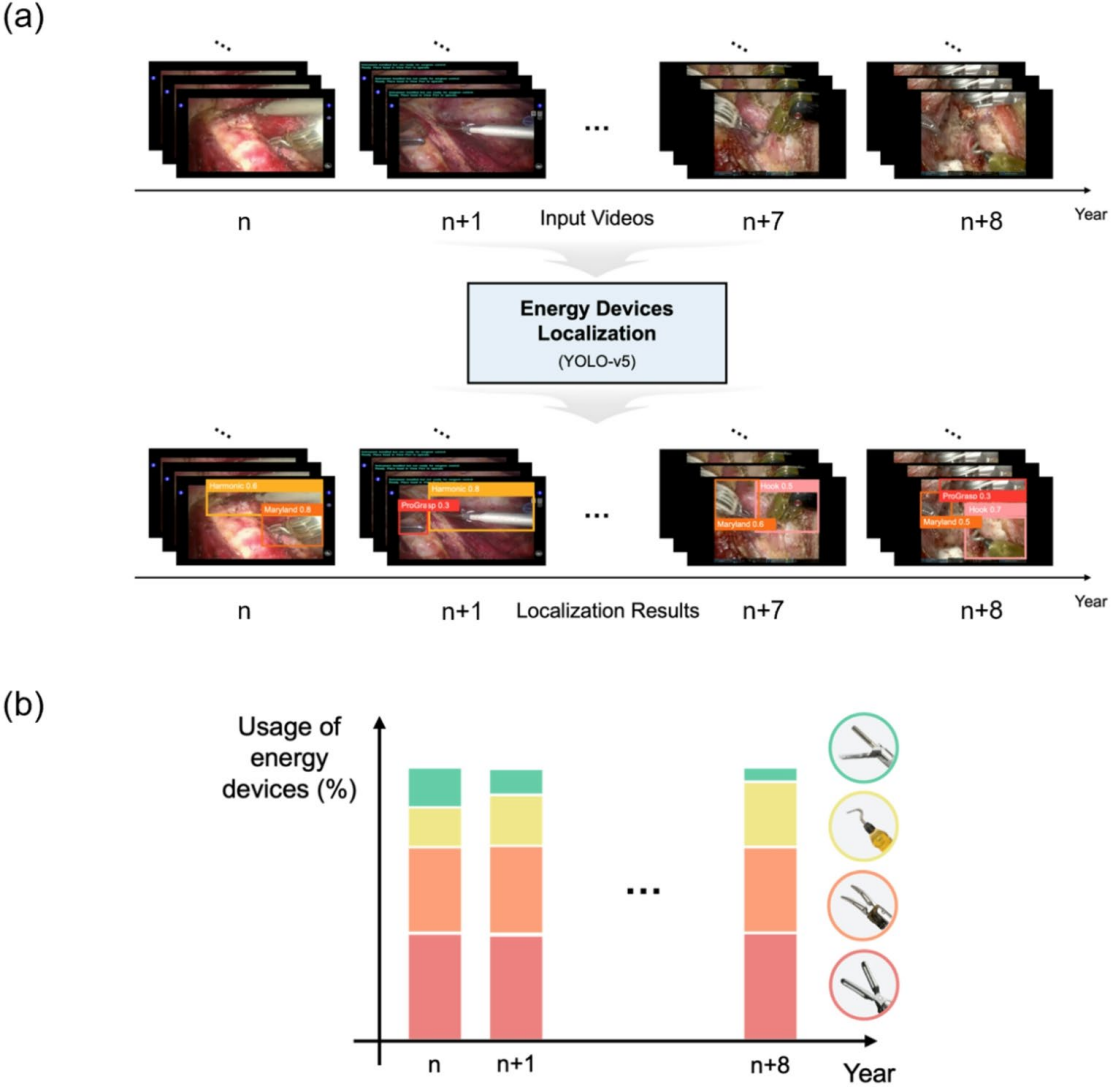
Overview of the study design. (**a**) The process used to identify energy devices. (**b**) Analysis of usage frequency (%)

This study retrospectively reviewed surgical videos from patients who underwent BABA RT for thyroid tumors at Seoul National University Bundang Hospital between January 2013 and December 2022. All procedures were performed by a single expert surgeon who has performed surgeries on more than 500 patients annually over the past 10 years. Only cases of right or left hemithyroidectomy were analyzed; cases of total thyroidectomy or modified radical neck dissection, as well as patients who had two or more concurrent procedures for other diseases, were excluded. From the high number of surgical patients, nine per year (from 2013 to 2021) were selected at random (i.e., *n* = 81). Then, the deep learning-based localization algorithm YOLOv5 [13] was used to analyze video footage, detect the type of energy device used automatically, and determine the annual usage frequency of each device (Fig. 1). The study protocol was approved by the Institutional Review Board of Seoul National University Bundang Hospital (B-2507-983-101), and consents were waived due to the retrospective nature of the study.

### Video sampling and annotation

The algorithm first assessed the frequency with which each of the following energy devices was used during surgery: ProGrasp Forceps, Maryland Bipolar Forceps, Harmonic ACE curved shears, and a Permanent cautery hook (Figure S1). In each video, the primary operative segment from midline incision to midline closure was defined by an endocrine surgeon. Only these clips were analyzed. The average video length was 32$\:\pm\:$19 min, and the resolution was 1,280 × 720 pixels. The videos had a frame rate of 30 frames per second, and the frames were sampled every 10 s. The sampled images were then normalized to pixel values (from 0 to 1) and resized to 416 × 416 pixels. Initially, all labels were annotated in Robotflow [14] by one board-certified surgeon, and then double-checked by a second to ensure inter-rater agreement.

### Model development and evaluation

The YOLOv5 object detection model [13] was trained on a separate dataset comprising 1,583 training images and 1,366 validation images. Its performance was then validated using 90 distinct test images. Assessment of model performance was based on the mean average precision (mAP) at an IoU threshold of 0.50 (mAP@50), averaged over thresholds from 0.50 to 0.95 (mAP@50–95). Class-specific precision and recall were also evaluated.

### Analysis of usage frequency and implementation

For each year, the frequency with which each device was used was calculated as the number of sampled frames in which that device was detected, divided by the total number of sampled frames. Trends over the 9-year period were visualized using line plots. The software was implemented in Python (ver. 3.8), incorporating the OpenCV (ver. 4.9.0), and PyTorch (ver. 1.8) packages. The deep learning model was trained for 100 epochs, with a batch size of 12, using the Adam optimizer and a learning rate of 0.00001. All training and inference procedures were executed on a T4 GPU server.

## Results

### Patient demographics

Eighty-one patients were included in the study; the mean age was 38.8 (± 9.6) years (Table 1). The cohort showed a significant female predominance (86.2%; *n* = 69). The mean body mass index was 22.9 (± 3.5). The total percentage of cases undergoing right lobectomy was 55.6% (*n* = 45). Pathology was dominated overwhelmingly by papillary thyroid carcinoma (87.7%, *n* = 71). The mean tumor size was 1.28 (± 1.23) cm. Less common diagnoses included follicular thyroid carcinoma, follicular adenoma, and nodular hyperplasia, each accounting for less than 5% of the total cases.

**Table 1:** Patient demographics

Variable		Percentage
Total (n)	81	
Age (yr), mean ± SD	38.8 (± 9.6)	
Sex		
Male	11	13.8%
Female	69	86.2%
BMI, mean ± SD	22.9 (± 3.5)	
Main operative type		
Rt. Lobectomy	45	55.6%
Lt. Lobectomy	36	44.4%
Tumor size (cm)	1.28 (± 1.23)	
Pathology		
Papillary thyroid carcinoma	71	87.7%
Follicular thyroid carcinoma	2	2.5%
Follicular adenoma	4	4.9%
Nodular hyperplasia	4	4.9%

SD, standard deviation; Rt, right; Lt, left; yr, years

## Results of energy device localization

Table 2 presents a quantitative summary of the performance of the YOLOv5 model with respect to identifying a particular energy device. Overall, the model achieved strong results, with a precision of 0.835, a recall of 0.819, a mAP@50 of 0.887, and a mAP@50–95 of 0.646.

**Table 2:** Quantitative performance of the algorithm for identifying an energy device

Energy device	Precision	Recall	mAP@50	mAP@50–95
ProGrasp forceps	0.834	0.777	0.867	0.618
Permanent cautery hook	0.719	0.894	0.882	0.657
Maryland bipolar forceps	0.834	0.835	0.881	0.645
Harmonic ACE curved shears	0.954	0.770	0.918	0.667
Average	0.835	0.819	0.887	0.646

*mAP, mean Average Precision

**Fig. 2:**
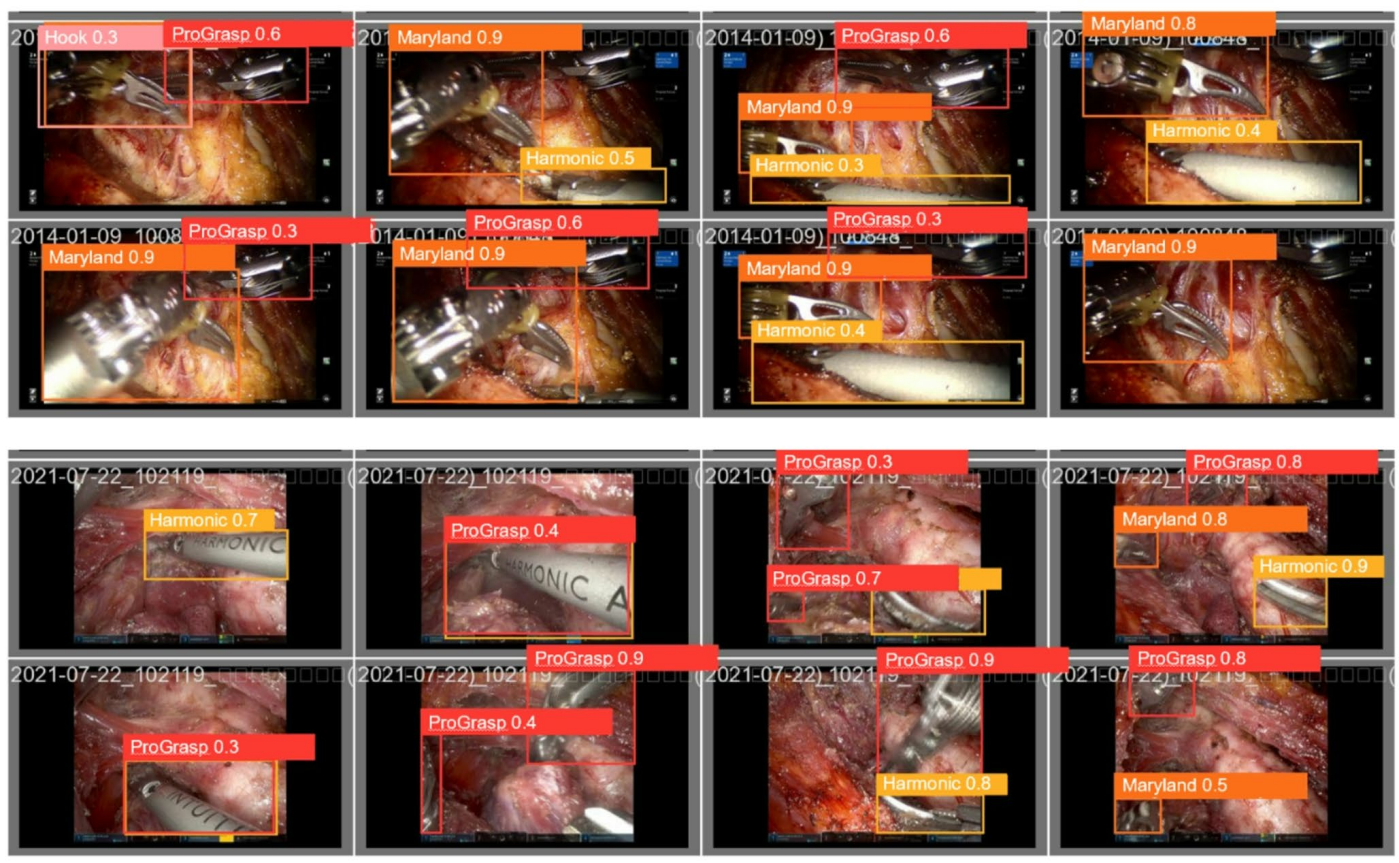
Qualitative results of energy device localization. The figure shows examples in which the algorithm identified the detected device, along with the corresponding probability values calculated by the detection model. Hook (Permanent cautery hook), Harmonic (Harmonic ACE curved shears), Maryland (Maryland bipolar forceps) and ProGrasp (ProGrasp forceps)

When broken down by instrument, the data showed that the ProGrasp forceps and Maryland bipolar forceps had near-identical detection performance, with a precision of 0.834 and 0.835, and a recall of 0.777 and 0.835, respectively. The Permanent cautery hook achieved the highest recall (0.894), while the Harmonic ACE curved shears demonstrated exceptional accuracy (precision, 0.954). These results confirm that YOLOv5 can reliably identify a range of energy devices used during surgery. A visual representation of these results is provided in Fig. 2.

### Temporal trends in the use of different energy devices

**Fig. 3:**
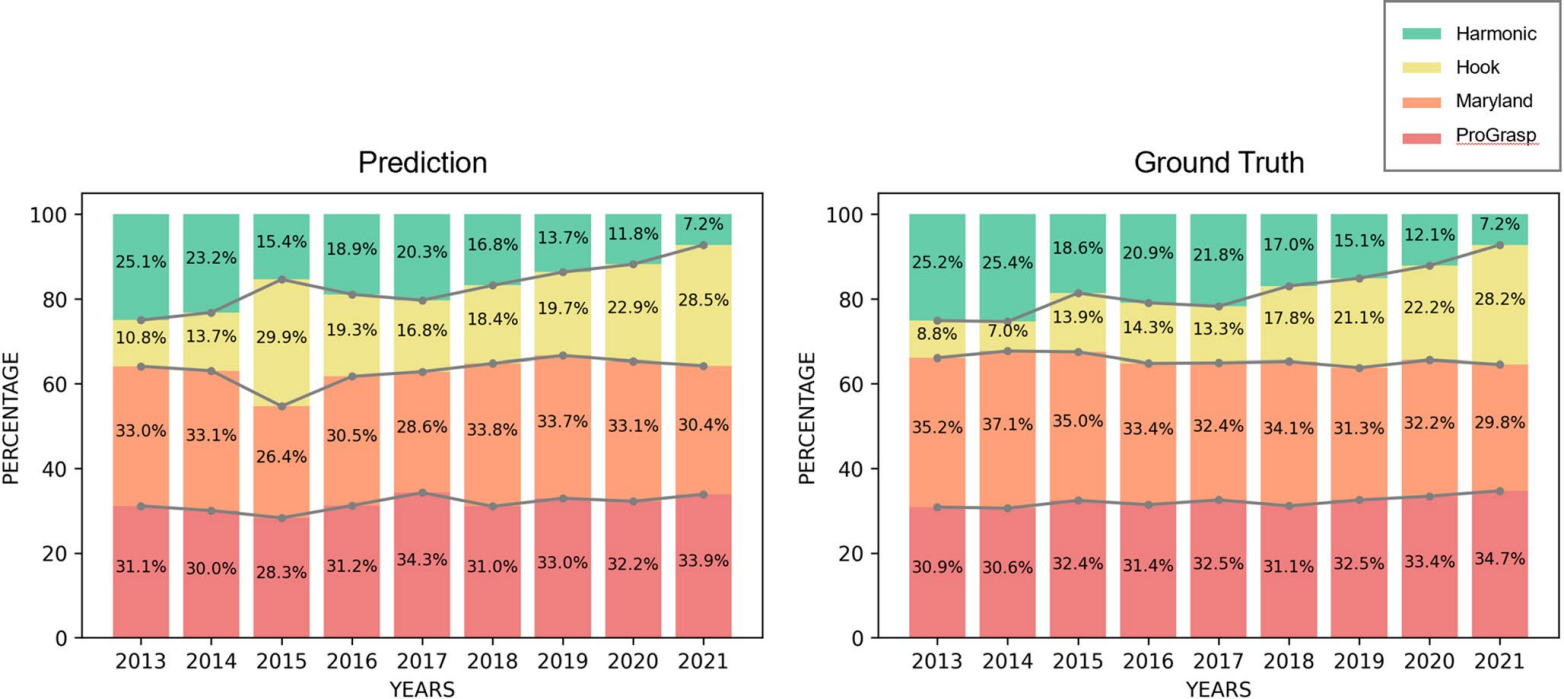
Yearly change in the predicted use (expressed as a percentage) of energy devices based on analysis by the algorithm (data from 2013–2021)

Figure 3 clearly illustrates a decade-long shift in the preference for energy devices. From 2013 to 2021, use of the Permanent cautery hook increased markedly (by 51.6%), while use of the Harmonic ACE curved shears decreased by the same percentage. In addition, use of ProGrasp forceps rose by 3.8%, whereas that of Maryland bipolar forceps declined by 5.4%. Notably, use of a Permanent cautery hook increased by 19.4%, whereas that of Harmonic ACE curved shears dropped by 18.0%. Statistically, the ability to predict use of an particular energy device was statistically comparable with the ground truth ($\:p>0.05)$, with the only exception being use of the Harmonic ACE curved shears and the Permanent cautery hook in 2015.

## Discussion

This retrospective, video-based analysis of 81 BABA RT cases spanning nine years used the YOLOv5 object detection algorithm to automatically quantify use of four different energy devices during BABA RT. We observed a notable shift in device use from 2013 to 2021: use of Harmonic ACE curved shears fell significantly from 72.3% in 2013 to 20.7% in 2021, while that of a Permanent cautery hook increased from 27.7% to 79.3% over the same period. By contrast, use of ProGrasp and Maryland bipolar forceps remained relatively stable throughout the study period. Taken together, these data reveal that surgical preferences and techniques for BABA RT have evolved over the past decade.

In the early days of robotic surgery, there was a heavy reliance on the Harmonic ACE to ensure stability during hemostasis and reduce postoperative bleeding; however, as surgeons gained experience, they realized that Permanent cautery hooks were also sufficient for hemostasis. Furthermore, the Harmonic ACE is a straight device that cannot be bent, whereas the Permanent cautery hook is able to bend, thereby offering a marked advantage. This freedom of motion has likely contributed to its increasing use by surgeons. Furthermore, the Permanent cautery hook also offers an advantage with respect to heat transfer to surrounding tissue. It is the combination of all of these factors that likely contributed to the increasing use of the Permanent cautery hook.

Despite the rapid development of robots, deep learning-based techniques have not yet been widely adopted in everyday robotic surgery. This limited utilization is largely due to practical, rather than methodological, problems. During surgery, deployment requires rigorous real-time performance, high robustness against unexpected visual changes, seamless integration with robotic systems, and clear evidence of clinical benefits, which presents significant obstacles to a wide range of implementations. Therefore, we focused on retrospective and automated video analyses to objectively quantify the long-term trends in surgical practice. This study bypasses many barriers related to real-time clinical deployment by positioning deep learning as an analytical tool rather than an interventional tool. In this context, our findings show that artificial intelligence can be practically utilized to study the evolution of large-scale surgical techniques, providing insights that are difficult to obtain from manual review alone.

This research has several limitations. First, this was a single-center, single‐surgeon study, meaning that generalizability of the findings to other institutions and diverse surgical practices is limited. Second, while the YOLOv5 model [13] achieved high overall accuracy, misclassification of small or partially occluded energy devices may have introduced measurement errors, meaning that our reported usage frequencies represent broad trends rather than exact dwell times. Third, we did not perform formal statistical trend analyses or report confidence intervals, so the year‐to‐year statistical significance of the observed changes remains untested. Finally, we did not account for unmeasured confounders such as changes in patient case mix, tumor characteristics, or ancillary equipment, any of which could have influenced device selection independently of surgeon preference.

Future work should validate this automated video-analysis pipeline across multiple centers, using a broader cohort of surgeons to determine whether the observed shift from Harmonic ACE curved shears to a Permanent cautery hook is a universal trend. Integrating clinical outcome data such as operative time, blood loss, and complication rates will be essential to establish whether changes in device use translate into tangible benefits for patients. Furthermore, expanding the detection model to include additional energy devices (e.g., clips or suturing devices), as well as segmenting distinct operative phases, will enable more granular workflow analyses. Finally, incorporating formal statistical methods for trend testing, and adjusting for potential confounders, will strengthen the robustness and interpretability of future longitudinal studies.

## Conclusions

Here, we demonstrate that a deep learning-based algorithm was able to automatically track trends in use of energy devices for BABA RT. The findings suggest a significant shift from use of Harmonic ACE curved shears to that of a Permanent cautery hook over the past decade, reflecting evolving surgical preferences and techniques.

## Supplementary Information

Below is the link to the electronic supplementary material.

Supplementary Material 1

## Data Availability

No datasets were generated or analysed during the current study.
