# Supplementary material for: A decade-long shift in use of energy devices for BABA robotic thyroidectomy: automated video analysis by deep learning
Source: J Robot Surg. 2026 Mar 17;20(1):356. doi: 10.1007/s11701-025-03113-7 (PMC12995972; doi:10.1007/s11701-025-03113-7)
Supplement: Supplementary file 1 — Supplementary Material 1 [file 11701_2025_3113_MOESM1_ESM.docx]

**Supplementary Materials**

**
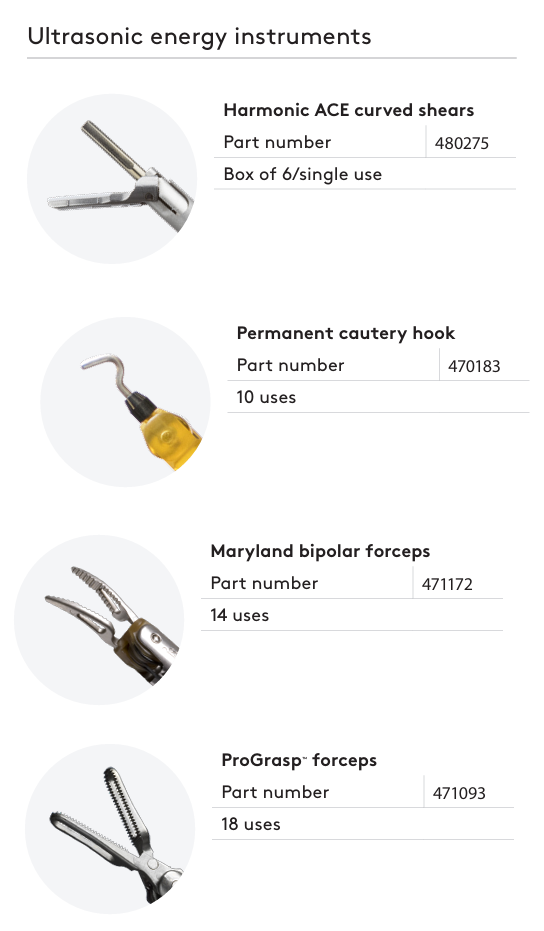
**

Figure S1. Example of energy devices: Harmonic ACE curved shears, Permanent cautery hook, Maryland bipolar forceps and ProGrasp forceps. Reproduced under the terms of the Creative Commons Attribution 4.0 International License (CC BY 4.0; https://creativecommons.org/licenses/by/4.0/).
